# Bis[2-(benzyl­idene­amino)­phen­yl] disulfide

**DOI:** 10.1107/S1600536811048239

**Published:** 2011-11-19

**Authors:** Yong Wang, Shanshan Shi, Yanhua Han, Guo-Dong Wei

**Affiliations:** a Department of Chemistry, Liaocheng University, Liaocheng 252059, People’s Republic of China; b Liaocheng Labor Technical Schools, Liaocheng 252059, People’s Republic of China; c Shandong Donge Experimental High School, Donge, Shandong Province 252200, People’s Republic of China

## Abstract

In the title mol­ecule, C_26_H_20_N_2_S_2_, the two benzene rings connected by a disulfide chain form a dihedral angle of 84.9 (1)°, and the two benzene rings in the two benzyl­idene­amino­phenyl fragments form dihedral angles of 34.4 (1) and 32.8 (1)°. The crystal structure exhibits weak inter­molecular C—H⋯S hydrogen bonds, which link the mol­ecules into chains along [101].

## Related literature

For general background to Schiff bases and their synthesis, see: Wang *et al.* (1998 ▶); Bai *et al.* (2005 ▶). For a related structure, see: He *et al.* (2011 ▶).
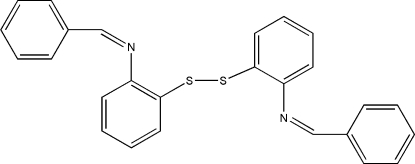


## Experimental

### 

#### Crystal data

C_26_H_20_N_2_S_2_
*M* *_r_* = 424.56
Monoclinic, 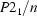

*a* = 10.2421 (11) Å
*b* = 19.672 (2) Å
*c* = 11.4739 (13) Å
β = 97.198 (1)°
*V* = 2293.5 (4) Å^3^
*Z* = 4
Mo *K*α radiation
μ = 0.25 mm^−1^
*T* = 298 K
0.43 × 0.35 × 0.31 mm

#### Data collection

Bruker SMART APEX CCD area-etector diffractometer
Absorption correction: multi-scan (*SADABS*; Sheldrick, 1996 ▶) *T*_min_ = 0.901, *T*_max_ = 0.927
11618 measured reflections
4043 independent reflections
1764 reflections with *I* > 2σ(*I*)
*R*_int_ = 0.125

#### Refinement

*R*[*F* ^2^ > 2σ(*F* ^2^)] = 0.060
*wR*(*F* ^2^) = 0.197
*S* = 1.05
4043 reflections
271 parameters
H-atom parameters constrained
Δρ_max_ = 0.27 e Å^−3^
Δρ_min_ = −0.24 e Å^−3^

### 

Data collection: *SMART* (Bruker, 2007 ▶); cell refinement: *SAINT* (Bruker, 2007 ▶); data reduction: *SAINT*; program(s) used to solve structure: *SHELXS97* (Sheldrick, 2008 ▶); program(s) used to refine structure: *SHELXL97* (Sheldrick, 2008 ▶); molecular graphics: *SHELXTL* (Sheldrick, 2008 ▶); software used to prepare material for publication: *SHELXTL*.

## Supplementary Material

Crystal structure: contains datablock(s) I, global. DOI: 10.1107/S1600536811048239/cv5194sup1.cif

Structure factors: contains datablock(s) I. DOI: 10.1107/S1600536811048239/cv5194Isup2.hkl

Supplementary material file. DOI: 10.1107/S1600536811048239/cv5194Isup3.cml

Additional supplementary materials:  crystallographic information; 3D view; checkCIF report

## Figures and Tables

**Table 1:** Hydrogen-bond geometry (Å, °)

*D*—H⋯*A*	*D*—H	H⋯*A*	*D*⋯*A*	*D*—H⋯*A*
C5—H5⋯S1^i^	0.93	2.86	3.604 (5)	137

Symmetry code: (i) 
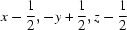
.

**Fig. 1.**
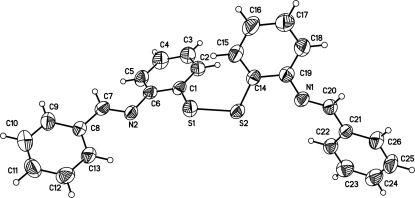
The title molecule with the atomic numbering scheme. The displacement ellipsoids are shown at the 30% probability level.

## supplementary crystallographic information

### Comment

Schiff bases  have received considerable attention during the last decades, mainly due to their coordinative and electronic properties (Wang *et al.*, 1998). For this reason, much effort has been devoted to develop effcient routes for the synthesis of these classes of compounds (Bai *et al.*, 2005). In this paper, we report the crystal structure of the title compound, (I), obtained by the reaction of  benzaldehyde and 2,2'-diaminodiphenyl disulfide.

In (I) (Fig. 1), the molecule has a *trans* configuration about the S—S bond. The bond lengths an angles are normal  and are comparable to the values observed in N,N'-bis(4-(dimethylamino)benzylidene)-2,2'-diaminodiphenyl disulfide  (He *et al.*, 2011). Two benzene rings connected through disulfide chain form a dihedral angle of 84.9 (1)°, and two benzene rings in two benzylideneaniline fragments form the dihedral angles of 34.4 (1) and 32.8 (1)°, respectively.

The crystal packing of the title compound exhibits weak intermolecular C—H···S hydrogen bonds (Table 1), which link molecules into chains along [101].

### Experimental

A mixture of benzaldehyde (10 mol), 2,2'-diaminodiphenyl disulfide (5 mol) was refluxed in 20 mL of ethanol for 3.0 hrs. The reaction completion was monitored through thin layer chromatography and the reaction mixture was cooled to room tempertature . The precipitate obtained was filtered, dried and crystallized from ethanol to obtain the title compound.

### Refinement

All H atoms were placed in geometrically idealized positions (C-H 0.93 Å ) and treated as riding on their parent atoms, with U_iso_(H) = 1.2U_eq_(C).

### Crystal data

**Table:**

C_26_H_20_N_2_S_2_	*F*(000) = 888
*M**_r_* = 424.56	*D*_x_ = 1.230 Mg m^−^^3^
Monoclinic, *P*2_1_/*n*	Mo *K*α radiation, λ = 0.71073 Å
*a* = 10.2421 (11) Å	Cell parameters from 2078 reflections
*b* = 19.672 (2) Å	θ = 2.7–20.1°
*c* = 11.4739 (13) Å	µ = 0.25 mm^−^^1^
β = 97.198 (1)°	*T* = 298 K
*V* = 2293.5 (4) Å^3^	Block, yellow
*Z* = 4	0.43 × 0.35 × 0.31 mm

### Data collection

**Table:**

Bruker SMART APEX CCD area-etector diffractometer	4043 independent reflections
Radiation source: fine-focus sealed tube	1764 reflections with *I* > 2σ(*I*)
graphite	*R*_int_ = 0.125
φ and ω scans	θ_max_ = 25.0°, θ_min_ = 2.7°
Absorption correction: multi-scan (*SADABS*; Sheldrick, 1996)	*h* = −12→12
*T*_min_ = 0.901, *T*_max_ = 0.927	*k* = −20→23
11618 measured reflections	*l* = −13→11

### Refinement

**Table:**

Refinement on *F*^2^	Primary atom site location: structure-invariant direct methods
Least-squares matrix: full	Secondary atom site location: difference Fourier map
*R*[*F*^2^ > 2σ(*F*^2^)] = 0.060	Hydrogen site location: inferred from neighbouring sites
*wR*(*F*^2^) = 0.197	H-atom parameters constrained
*S* = 1.05	*w* = 1/[σ^2^(*F*_o_^2^) + (0.0425*P*)^2^ + 0.9182*P*] where *P* = (*F*_o_^2^ + 2*F*_c_^2^)/3
4043 reflections	(Δ/σ)_max_ < 0.001
271 parameters	Δρ_max_ = 0.27 e Å^−^^3^
0 restraints	Δρ_min_ = −0.24 e Å^−^^3^

### Special details

**Table:**

Geometry. All esds (except the esd in the dihedral angle between two l.s. planes) are estimated using the full covariance matrix. The cell esds are taken into account individually in the estimation of esds in distances, angles and torsion angles; correlations between esds in cell parameters are only used when they are defined by crystal symmetry. An approximate (isotropic) treatment of cell esds is used for estimating esds involving l.s. planes.
Refinement. Refinement of F^2^ against ALL reflections. The weighted R-factor wR and goodness of fit S are based on F^2^, conventional R-factors R are based on F, with F set to zero for negative F^2^. The threshold expression of F^2^ > 2sigma(F^2^) is used only for calculating R-factors(gt) etc. and is not relevant to the choice of reflections for refinement. R-factors based on F^2^ are statistically about twice as large as those based on F, and R- factors based on ALL data will be even larger.

### Fractional atomic coordinates and isotropic or equivalent isotropic displacement parameters (Å^2^)

**Table:**

	*x*	*y*	*z*	*U*_iso_*/*U*_eq_	
N1	0.3113 (4)	0.0052 (2)	0.2975 (3)	0.0710 (11)	
N2	−0.1878 (4)	0.2777 (2)	0.1398 (3)	0.0691 (11)	
S1	0.03931 (13)	0.20245 (7)	0.22438 (11)	0.0799 (5)	
S2	0.13336 (13)	0.11360 (7)	0.27203 (11)	0.0730 (5)	
C1	−0.0581 (4)	0.1836 (2)	0.0887 (4)	0.0634 (13)	
C2	−0.0300 (5)	0.1322 (3)	0.0121 (4)	0.0793 (15)	
H2	0.0438	0.1049	0.0310	0.095*	
C3	−0.1116 (6)	0.1215 (3)	−0.0918 (5)	0.0934 (18)	
H3	−0.0924	0.0871	−0.1427	0.112*	
C4	−0.2203 (6)	0.1613 (3)	−0.1198 (5)	0.1015 (19)	
H4	−0.2759	0.1534	−0.1890	0.122*	
C5	−0.2480 (5)	0.2136 (3)	−0.0457 (4)	0.0833 (16)	
H5	−0.3215	0.2409	−0.0659	0.100*	
C6	−0.1665 (5)	0.2255 (3)	0.0588 (4)	0.0660 (13)	
C7	−0.2390 (5)	0.3338 (3)	0.1026 (4)	0.0725 (14)	
H7	−0.2612	0.3392	0.0220	0.087*	
C8	−0.2650 (5)	0.3901 (3)	0.1794 (5)	0.0715 (14)	
C9	−0.3206 (5)	0.4488 (3)	0.1287 (6)	0.0952 (18)	
H9	−0.3408	0.4520	0.0476	0.114*	
C10	−0.3455 (6)	0.5022 (4)	0.1994 (8)	0.120 (2)	
H10	−0.3823	0.5418	0.1654	0.144*	
C11	−0.3178 (7)	0.4986 (3)	0.3175 (8)	0.111 (2)	
H11	−0.3384	0.5349	0.3639	0.133*	
C12	−0.2592 (6)	0.4413 (3)	0.3693 (6)	0.1038 (19)	
H12	−0.2372	0.4391	0.4503	0.125*	
C13	−0.2333 (6)	0.3868 (3)	0.2991 (5)	0.0928 (17)	
H13	−0.1942	0.3478	0.3333	0.111*	
C14	0.2829 (5)	0.1152 (2)	0.2100 (4)	0.0651 (13)	
C15	0.3270 (6)	0.1690 (3)	0.1459 (5)	0.0845 (16)	
H15	0.2761	0.2082	0.1344	0.101*	
C16	0.4451 (7)	0.1652 (3)	0.0993 (6)	0.115 (2)	
H16	0.4732	0.2015	0.0569	0.138*	
C17	0.5212 (6)	0.1068 (4)	0.1163 (7)	0.128 (3)	
H17	0.6001	0.1035	0.0843	0.153*	
C18	0.4787 (6)	0.0531 (3)	0.1814 (6)	0.108 (2)	
H18	0.5296	0.0139	0.1924	0.130*	
C19	0.3620 (5)	0.0573 (3)	0.2299 (4)	0.0732 (14)	
C20	0.3876 (5)	−0.0331 (3)	0.3620 (5)	0.0722 (14)	
H20	0.4779	−0.0266	0.3646	0.087*	
C21	0.3406 (6)	−0.0872 (2)	0.4330 (4)	0.0667 (13)	
C22	0.2066 (6)	−0.1007 (3)	0.4311 (5)	0.0795 (15)	
H22	0.1453	−0.0757	0.3819	0.095*	
C23	0.1646 (6)	−0.1507 (3)	0.5016 (6)	0.0992 (18)	
H23	0.0752	−0.1598	0.4990	0.119*	
C24	0.2532 (9)	−0.1871 (3)	0.5753 (6)	0.104 (2)	
H24	0.2237	−0.2198	0.6243	0.125*	
C25	0.3835 (9)	−0.1760 (3)	0.5775 (6)	0.111 (2)	
H25	0.4436	−0.2019	0.6263	0.134*	
C26	0.4280 (6)	−0.1253 (3)	0.5061 (5)	0.0976 (18)	
H26	0.5178	−0.1175	0.5083	0.117*	

### Atomic displacement parameters (Å^2^)

**Table:**

	*U*^11^	*U*^22^	*U*^33^	*U*^12^	*U*^13^	*U*^23^
N1	0.088 (3)	0.058 (3)	0.067 (3)	−0.001 (2)	0.009 (2)	0.006 (2)
N2	0.087 (3)	0.063 (3)	0.056 (2)	0.008 (2)	0.001 (2)	0.007 (2)
S1	0.0979 (10)	0.0781 (10)	0.0572 (8)	0.0153 (7)	−0.0157 (7)	−0.0105 (7)
S2	0.0825 (9)	0.0791 (9)	0.0549 (7)	0.0065 (7)	−0.0016 (6)	0.0091 (7)
C1	0.074 (3)	0.065 (3)	0.048 (3)	0.000 (3)	−0.005 (2)	0.005 (3)
C2	0.097 (4)	0.081 (4)	0.058 (3)	0.014 (3)	0.000 (3)	−0.006 (3)
C3	0.128 (5)	0.089 (4)	0.057 (3)	0.015 (4)	−0.011 (3)	−0.017 (3)
C4	0.128 (5)	0.105 (5)	0.061 (3)	0.013 (4)	−0.028 (3)	−0.016 (4)
C5	0.096 (4)	0.086 (4)	0.061 (3)	0.013 (3)	−0.017 (3)	0.005 (3)
C6	0.081 (3)	0.069 (3)	0.046 (3)	0.001 (3)	0.001 (2)	0.000 (3)
C7	0.083 (3)	0.076 (4)	0.057 (3)	0.001 (3)	0.005 (3)	0.011 (3)
C8	0.083 (3)	0.061 (3)	0.071 (4)	0.005 (3)	0.013 (3)	0.008 (3)
C9	0.109 (4)	0.078 (4)	0.096 (4)	0.022 (3)	0.006 (4)	0.009 (4)
C10	0.133 (6)	0.087 (5)	0.139 (7)	0.033 (4)	0.020 (5)	0.005 (5)
C11	0.131 (5)	0.073 (5)	0.137 (7)	0.008 (4)	0.052 (5)	−0.013 (5)
C12	0.153 (6)	0.079 (4)	0.082 (4)	−0.015 (4)	0.026 (4)	−0.002 (4)
C13	0.140 (5)	0.059 (4)	0.083 (4)	0.007 (3)	0.028 (4)	0.009 (3)
C14	0.071 (3)	0.062 (3)	0.060 (3)	0.000 (3)	−0.002 (2)	0.004 (3)
C15	0.091 (4)	0.072 (4)	0.089 (4)	0.004 (3)	0.004 (3)	0.022 (3)
C16	0.109 (5)	0.103 (5)	0.136 (6)	0.004 (4)	0.027 (5)	0.057 (5)
C17	0.104 (5)	0.135 (6)	0.155 (7)	0.014 (5)	0.053 (5)	0.059 (6)
C18	0.104 (5)	0.093 (5)	0.132 (6)	0.020 (4)	0.038 (4)	0.038 (4)
C19	0.071 (3)	0.075 (4)	0.072 (3)	0.000 (3)	0.003 (3)	0.008 (3)
C20	0.080 (3)	0.063 (3)	0.074 (3)	0.006 (3)	0.011 (3)	0.001 (3)
C21	0.090 (4)	0.053 (3)	0.057 (3)	0.008 (3)	0.008 (3)	−0.003 (3)
C22	0.099 (4)	0.058 (3)	0.082 (4)	0.003 (3)	0.013 (3)	0.006 (3)
C23	0.118 (5)	0.076 (4)	0.104 (5)	−0.013 (4)	0.019 (4)	0.005 (4)
C24	0.171 (7)	0.054 (4)	0.089 (5)	0.002 (4)	0.021 (5)	0.008 (3)
C25	0.157 (7)	0.075 (4)	0.101 (5)	0.042 (5)	0.008 (5)	0.020 (4)
C26	0.115 (5)	0.082 (4)	0.095 (4)	0.024 (4)	0.011 (4)	0.019 (4)

### Geometric parameters (Å, °)

**Table:**

N1—C20	1.258 (6)	C12—C13	1.386 (7)
N1—C19	1.423 (6)	C12—H12	0.9300
N2—C7	1.272 (5)	C13—H13	0.9300
N2—C6	1.420 (6)	C14—C15	1.395 (7)
S1—C1	1.779 (4)	C14—C19	1.400 (6)
S1—S2	2.0371 (18)	C15—C16	1.385 (7)
S2—C14	1.768 (5)	C15—H15	0.9300
C1—C6	1.391 (6)	C16—C17	1.389 (8)
C1—C2	1.394 (6)	C16—H16	0.9300
C2—C3	1.383 (7)	C17—C18	1.394 (8)
C2—H2	0.9300	C17—H17	0.9300
C3—C4	1.367 (7)	C18—C19	1.383 (7)
C3—H3	0.9300	C18—H18	0.9300
C4—C5	1.386 (7)	C20—C21	1.458 (7)
C4—H4	0.9300	C20—H20	0.9300
C5—C6	1.393 (6)	C21—C26	1.371 (7)
C5—H5	0.9300	C21—C22	1.395 (7)
C7—C8	1.461 (7)	C22—C23	1.377 (7)
C7—H7	0.9300	C22—H22	0.9300
C8—C13	1.372 (7)	C23—C24	1.363 (8)
C8—C9	1.383 (7)	C23—H23	0.9300
C9—C10	1.370 (8)	C24—C25	1.349 (8)
C9—H9	0.9300	C24—H24	0.9300
C10—C11	1.351 (9)	C25—C26	1.402 (8)
C10—H10	0.9300	C25—H25	0.9300
C11—C12	1.377 (8)	C26—H26	0.9300
C11—H11	0.9300		
			
C20—N1—C19	120.7 (5)	C8—C13—H13	119.8
C7—N2—C6	119.9 (4)	C12—C13—H13	119.8
C1—S1—S2	104.50 (17)	C15—C14—C19	119.0 (5)
C14—S2—S1	106.31 (17)	C15—C14—S2	125.4 (4)
C6—C1—C2	119.7 (4)	C19—C14—S2	115.6 (4)
C6—C1—S1	115.8 (4)	C16—C15—C14	121.2 (5)
C2—C1—S1	124.5 (4)	C16—C15—H15	119.4
C3—C2—C1	120.3 (5)	C14—C15—H15	119.4
C3—C2—H2	119.9	C15—C16—C17	119.6 (6)
C1—C2—H2	119.9	C15—C16—H16	120.2
C4—C3—C2	120.1 (5)	C17—C16—H16	120.2
C4—C3—H3	119.9	C16—C17—C18	119.6 (6)
C2—C3—H3	119.9	C16—C17—H17	120.2
C3—C4—C5	120.3 (5)	C18—C17—H17	120.2
C3—C4—H4	119.8	C19—C18—C17	120.9 (6)
C5—C4—H4	119.8	C19—C18—H18	119.5
C4—C5—C6	120.4 (5)	C17—C18—H18	119.5
C4—C5—H5	119.8	C18—C19—C14	119.7 (5)
C6—C5—H5	119.8	C18—C19—N1	124.7 (5)
C1—C6—C5	119.1 (5)	C14—C19—N1	115.6 (5)
C1—C6—N2	116.8 (4)	N1—C20—C21	122.8 (5)
C5—C6—N2	124.1 (5)	N1—C20—H20	118.6
N2—C7—C8	123.6 (5)	C21—C20—H20	118.6
N2—C7—H7	118.2	C26—C21—C22	118.1 (5)
C8—C7—H7	118.2	C26—C21—C20	120.3 (5)
C13—C8—C9	119.6 (5)	C22—C21—C20	121.6 (5)
C13—C8—C7	122.0 (5)	C23—C22—C21	120.4 (5)
C9—C8—C7	118.4 (5)	C23—C22—H22	119.8
C10—C9—C8	119.2 (6)	C21—C22—H22	119.8
C10—C9—H9	120.4	C24—C23—C22	120.5 (6)
C8—C9—H9	120.4	C24—C23—H23	119.8
C11—C10—C9	121.6 (7)	C22—C23—H23	119.8
C11—C10—H10	119.2	C25—C24—C23	120.4 (6)
C9—C10—H10	119.2	C25—C24—H24	119.8
C10—C11—C12	120.0 (6)	C23—C24—H24	119.8
C10—C11—H11	120.0	C24—C25—C26	119.8 (6)
C12—C11—H11	120.0	C24—C25—H25	120.1
C11—C12—C13	119.2 (6)	C26—C25—H25	120.1
C11—C12—H12	120.4	C21—C26—C25	120.7 (6)
C13—C12—H12	120.4	C21—C26—H26	119.6
C8—C13—C12	120.4 (5)	C25—C26—H26	119.6

### Hydrogen-bond geometry (Å, °)

**Table:**

*D*—H···*A*	*D*—H	H···*A*	*D*···*A*	*D*—H···*A*
C5—H5···S1^i^	0.93	2.86	3.604 (5)	137.

Symmetry codes: (i) *x*−1/2, −*y*+1/2, *z*−1/2.
